# Robot-Assisted Pelvic Dissection for Enlarged Lymph Nodes in Melanoma Improves Recovery with Equivalent Oncological Outcomes to Open Pelvic Dissection

**DOI:** 10.1245/s10434-023-14834-0

**Published:** 2024-01-04

**Authors:** Amit Roshan, Bhumi Shah, Keith D. Anderson, Suzanne Murphy, Benjamin Thomas, Arthur S. McPhee, Benjamin W. Lamb, Amer J. Durrani, Animesh J. K. Patel

**Affiliations:** 1 Cancer Research UK Cambridge Institute, University of Cambridge, Cambridge, UK; 2 Department of Plastic Surgery, Cambridge University Hospitals NHS Trust, Cambridge, UK; 3 School of Clinical Medicine, University of Cambridge, Cambridge, UK; 4 Department of Urology, Cambridge University Hospitals NHS Trust, Cambridge, UK; 5 Present Address: Department of Surgery, Royal Melbourne Hospital, University of Melbourne and International Medical Robotics Academy, Melbourne, VIC Australia; 6 Present Address: Department of Urology, Barts Health NHS Trust, Barts Cancer Institute, Queen Mary University of London and University College London Hospitals, London, UK

## Abstract

**Background:**

Robot-assisted pelvic lymph node dissection (rPLND) has been reported in heterogenous groups of patients with melanoma, including macroscopic or at-high-risk-for microscopic metastasis. With changing indications for surgery in melanoma, and availability of effective systemic therapies, pelvic dissection is now performed for clinically detected bulky lymph node metastasis followed by adjuvant drug therapy. rPLND has not been compared with open pelvic lymph node dissection (oPLND) for modern practice.

**Methods:**

All patients undergoing pelvic node dissection for macroscopic melanoma at a single institution were reviewed as a cohort, observational study.

**Results:**

Twenty-two pelvic lymph node dissections were identified (8 oPLND; 14 rPLND). The number of pelvic lymph nodes removed was similar (median oPLND 6.5 (interquartile range [IQR] 6.0–12.5] versus rPLND 6.0 [3.75–9.0]), with frequent matted nodes (11/22, 50.0%). Operative time (median oPLND 130 min [IQR 95.5–182] versus rPLND 126 min [IQR 97.8–160]) and complications (Clavien-Dindo scale) were similar. Length of hospital stay (median 5.34 days (IQR 3.77–6.94) versus 1.98 days (IQR 1.39–3.50) and time to postoperative adjuvant therapy (median 11.6 weeks [IQR 10.6–18.5] versus 7.71 weeks [IQR 6.29–10.4]) were shorter in the rPLND group. No differences in pelvic lymph node recurrence (*p* = 0.984), distant metastatic recurrence (*p* = 0.678), or melanoma-specific survival (*p* = 0.655) were seen (median follow-up 21.1 months [rPLND] and 25.7 months [oPLND]).

**Conclusions:**

rPLND is an effective way to remove bulky pelvic lymph nodes in melanoma, with a shorter recovery and reduced interval to initiating adjuvant therapy compared with oPLND. This group of patients may especially benefit from neoadjuvant systemic approaches to management.

The management of pelvic lymph nodes in the context of primary cutaneous melanoma of the lower limbs or trunk continues to evolve with changes to surgical practice, effective systemic therapies, and improved imaging surveillance.

First, completion pelvic lymph node dissection for patients with microscopic melanoma identified through inguinal sentinel lymph node is no longer widespread after pivotal trials showed no survival benefit.^1,2^ National guidelines in most countries do not recommend completion lymph node dissection as a treatment strategy in the presence of sentinel lymph node-confirmed micrometastasis. The latest National Comprehensive Cancer Network (NCCN) practice guidelines recommends a pelvic dissection in the presence of radiologically detected iliac or obturator lymph nodes (Category 2A evidence), but recognises lower evidence for pelvic dissection in other indications, such as a positive Cloquet’s lymph node or ≥3 involved lymph nodes.^3^

Second, several international trials have confirmed the efficacy of adjuvant immunotherapy and targeted therapy in the presence of microscopic melanoma.^4–7^ This has led to the widescale adoption of drug-based adjuvant therapy in the presence of confirmed micrometastasis to the groin or pelvic lymph nodes identified on sentinel lymph node biopsy, especially with overall AJCC Stage ≥IIIB.

Finally, the increasing uptake of imaging-based surveillance in patients with Stage III or high-risk Stage II melanoma including PET/CT allows the identification of nodal relapse at an earlier time point.^3,8^ Neoadjuvant approaches in patients with nodal progression shows significant promise demonstrating improved nodal relapse-free and melanoma-specific survival in medium-term follow-up. Studies specifically in pelvic nodal recurrence have not been addressed.

Robot-assisted pelvic lymphadenectomy (rPLND) has previously been shown to perform well compared with traditional open pelvic lymphadenectomy (oPLND) in terms of shorter length of hospital stay and equivalent oncological outcomes.^9^ However, published experience of rPLND has been limited to mixed case series from one to ten and often focuses on the technical procedure without long-term oncological outcomes.^10–13^ A single, larger series of 22 rPLND cases has been reported with longer-term outcomes in melanoma, but this has been in a mixed cohort of patients, including those with microscopic melanoma and macroscopic melanoma, and dates from before effective adjuvant therapy being available for all patients.^9^

In this study, we sought to assess outcomes in a cohort of patients from a single academic centre who underwent rPLND for macroscopic melanoma and compare with a group who had oPLND at the same center with all patients planned for standard of care adjuvant systemic therapy.

## Methods

A retrospectively collected database was created for all patients undergoing a pelvic lymph node dissection for melanoma at Addenbrooke’s Hospital between the years 2012–2023. Patients were included if they were having rPLND or oPLND, in the context of biopsy-proven macroscopic melanoma lymph node involvement. All patients were treated with an intent for postoperative adjuvant therapy.

Cambridge University Hospitals NHS Trust is a large university cancer center that receives tertiary melanoma referrals from a population base of approximately 2.4 million people. All referrals are initially discussed at a melanoma multidisciplinary team meeting. All patients identified as part of this study were treated after appropriate informed surgical consent. Any inguinal lymph node dissection was performed by plastic surgeons with melanoma-specific experience, and the dissection extent was up to and including the Cloquet’s lymph node. The pelvic dissection (oPLND or rPLND) was undertaken by urology surgeons with appropriate robotic surgery technical experience.

Data were collected centrally through the hospital electronic medical record database (EPIC Systems Corporation, WI) and linked in with additional follow-up data from regional hospitals where indicated. Data abstracted included demographic characteristics, primary melanoma pathology, previous and ongoing melanoma treatments, and surgical details. The follow-up period was defined as the time between the pelvic lymph node dissection and the date of last clinical follow-up or death.

The study was registered with the Cambridge University Hospitals QSIS system (Project ID PRN11058) after institutional review.

### Pelvic Node Dissection

Pelvic dissection was performed by an open method until 2016, and a change was made to rPLND from 2017 onwards. Technical details of both oPLND and rPLND are well described in melanoma and other indications.^10,14^ Duration of pelvic dissection was calculated between the recorded knife-to-skin to the end-of-procedure recorded for the pelvic procedure only which was performed before any inguinal lymph node dissection if indicated. Total duration of the procedure was additionally recorded from knife-to-skin to the end-of-procedure time. Surgical complications were recorded according to the Clavien–Dindo scale.^15,16^

### Adjuvant Therapy

Systemic therapy was offered to all patients in both oPLND and rPLND groups, with either standard-of-care or on-trial targeted therapy (BRAF inhibitor monotherapy, or BRAF-MEK inhibitor combination therapy) or immunotherapy (CTLA-4 monotherapy, PD-1 monotherapy, or CTLA-4/PD-1 combination therapy). Pelvic external beam radiotherapy (48 Grays in 20 fractions) were given in the presence of extensive extracapsular spread or involved margins.^17^

### Statistical Analysis

Statistical analysis was conducted with GraphPad Prism^®^ version 10.0.3 (GraphPad Software LLC, San Diego, CA). Clinical or pathological variables were summarized using descriptive statistics. All continuous variables were treated as skewed. Continuous variables were presented as medians and interquartile range, whereas categorical variables were presented as frequencies and proportions. Characteristics between the oPLND and rPLND groups were compared by using the unpaired *t*-test for continuous variables, and chi-square test or chi-square test for trend for categorical values.

All time-to-event endpoints were calculated from the date of surgery. Recurrence-free survival (RFS) was calculated for both in-basin pelvic recurrence and any distant recurrence separately. Time interval was recorded from surgery until recurrence, death, or last follow-up (treated as censored). Melanoma-specific survival (MSS) was calculated from surgery until death due to melanoma or last follow-up date (treated as censored). Kaplan-Meier survival plots with log-rank tests were used to assess differences in RFS and MSS between oPLND and rPLND cohorts. Statistical tests were two-sided, and *p* < 0.05 was considered statistically significant.

## Results

**Fig. 1:**
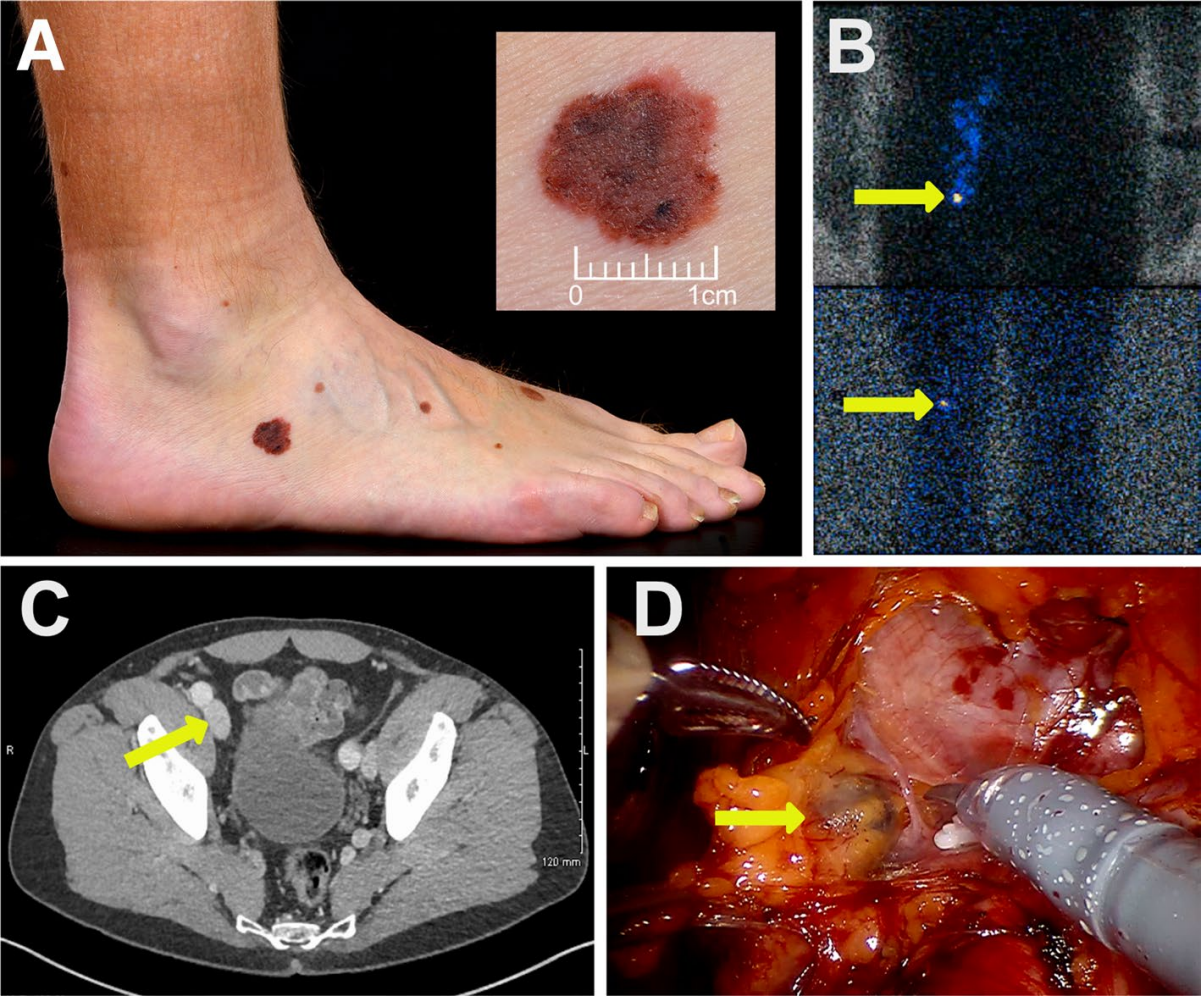
Treatment of pelvic lymph node macroscopic recurrence after primary cutaneous melanoma. **A** 52-year-old patient with BRAF V600E mutant pT1b melanoma on the right foot and no additional high-risk features. **B** Tc-99 Nanocolloid lymphoscintigram demonstrating sentinel lymph nodes in the right popliteal and right groin, both of which had no microscopic melanoma. **C** Clinical recurrence 7 years after initial presentation with inguinal lump and a staging CT scan demonstrating a 22-mm external iliac lymph node identified here. **D** Robot-assisted pelvic node dissection identifying the macroscopic lymph node adjacent to the external iliac vessels

Twenty-two patients underwent pelvic lymph node dissection for melanoma between November 2012 and October 2023. Eight patients had open pelvic lymph node dissection (oPLND) from 2012 to 2016, whereas 14 patients underwent robot-assisted pelvic lymph node dissection (rPLND) from 2017 to 2023. A typical patient in this cohort was a lower limb primary melanoma with macroscopic melanoma isolated lymph node progression several years after initial diagnosis (Fig. 1). Table 1 records the baseline demographic and primary melanoma pathological details, including sentinel lymph node status, where previously undertaken. Key determinants of melanoma behavior (age, tumor location, Breslow thickness, ulceration, mitotic index, and BRAF mutation status) were similar between the oPLND and rPLND group. The only significant difference between the two groups was the gender balance, where the rPLND group had significantly higher proportion of women compared to men (female oPLND 25.0% vs. rPLND 78.6%, *p* = 0.014). The pelvic disease-free interval between melanoma diagnosis and pelvic node identification was similar between the two groups (DFI oPLND median 2.5 years vs. rPLND 2.43 years, *p* = 0.440). Two patients in the oPLND group had melanoma of unknown primary.

**Table 1:** Demographic and tumour characteristics in patients according to method of pelvic lymph node surgery (open or robot-assisted)

	oPLND (*n* = 8) (2012–2016)	rPLND (*n* = 14) (2017–2023)	*p*^a^
Age at PLND (years)			0.643^b^
Median (IQR)	53.6 (48.1–57.0)	54.6 (45.8–69.6)	
Gender			0.014
Male	75.0 (n = 6)	21.4 (n = 3)	
Female	25.0 (n = 2)	78.6 (n = 11)	
Body mass index			0.556^b^
Median (IQR)	24.7 (22.0–36.5)	26.2 (20.9–31.1)	
ASA status			>0.999^c^
1	12.5 (n = 1)	14.3 (n = 2)	
2	75.0 (n = 6)	71.4 (n = 10)	
3	12.5 (n = 1)	14.3 (n = 2)	
Breslow thickness (mm)			0.917^b^
Median (IQR)	2.39 (1.10–4.53)	2.10 (1.10–3.95)	
Missing	2 (unknown primary)	1	
Ulceration			0.235
Yes	16.7 (n = 1)	45.4 (n = 5)	
No	83.3 (n = 5)	54.5 (n = 6)	
Missing	2	3	
Tumour mitotic rate			0.413^c^
0–1	0 (n = 0)	9.1 (n = 1)	
1–4	25.0 (n = 1)	36.4 (n = 4)	
≥ 5	75.0 (n = 3)	54.5 (n = 6)	
Missing	4	3	
Tumour site			0.142
Lower Limb	62.5 (n = 5)	78.6 (n = 11)	
Lower Trunk	12.5 (n = 1)	21.4 (n = 3)	
Unknown primary	25.0 (n = 2)	0	
BRAF mutation status			0.415
V600E mutant	62.5 (n = 5)	78.6 (n = 11)	
Wildtype	37.5 (n = 3)	21.4 (n = 3)	
NRAS mutation status			0.847
Q61R mutant	25.0 (n = 2)	21.4 (n = 3)	
Wildtype	75.0 (n = 6)	78.6 (n = 11)	
Sentinel lymph node status			0.251
Positive	25.0 (n = 2)	50.0 (n = 7)	
Negative or unknown	75.0 (n = 6)	50.0 (n = 7)	
Pelvic disease-free interval at diagnosis (years)			0.440^b^
Median (IQR)	2.5 (0.38–7.16)	2.43 (1.54–11.5)	

Values are percentages unless otherwise indicated. ^a^χ^2^ test; ^b^unpaired *t* test; ^c^χ^2^ for trend test

### Perioperative Details

There was no significant difference in preoperative determinants of outcome (age, body mass index (BMI), or American Society of Anesthesiologists (ASA) physical status) (Table 1).^18^

In the rPLND group, four patients underwent a pelvic dissection alone without an inguinal lymph node dissection, which was not different to the oPLND group (rPLND pelvic dissection alone 28.6% versus oPLND pelvic dissection alone 37.5%, *p* = 0.665; Table 2). Quality assurance metrics for adequacy of lymph node clearance, such as total number of lymph nodes, number of pelvic lymph nodes, and number of involved lymph nodes identified were similar in the treatment groups (Table 2). Pelvic dissection with either surgical approach was well tolerated with only two patients reporting Clavien-Dindo Grade IIIa complication in the oPLND group and one patient in the rPLND group. The IIIa complications were all readmissions related to drainage of pelvic collections requiring either radiological or surgical drain placement. There was one patient with wound dehiscence requiring surgical application of negative pressure dressings to achieve wound healing in the oPLND group. Qualitatively, the most common complications were seromas (oPLND 3/8 cases 37.5% and rPLND 6/14 cases 42.8%) and lymphoedema (oPLND 5/8 cases 62.5% and rPLND 6/14 cases 42.8%). There were no statistical differences in the rate of postsurgical complications reported overall between the two groups (*p* = 0.699; Table 2).

**Table 2:** Operative characteristics and perioperative outcomes in patients according to method of pelvic lymph node surgery (open or robot-assisted approach)

	oPLND (*n* = 8) (2012–2016)	rPLND (*n* = 14) (2017–2023)	*p*^a^
Extent of surgery			0.665
PLND alone (no inguinal LND)	37.5 (n = 3)	28.6 (n = 4)	
Inguinal LND + PLND	62.5 (n = 5)	71.4 (n = 10)	
Total operative time, min			0.335^b^
Median (IQR)	174 (158–216)	250 (160–303)	
Total pelvic dissection time, min			0.836^b^
Median (IQR)	130 (95.5–182)	126 (97.8–161)	
Total groin dissection time, min^*^			0.036^b^
Median (IQR)	72 (69.5–103)	112 (83–117)	
Length of inpatient stay, days^†^			0.006^b^
Median (IQR)	5.34 (3.77–6.94)	1.98 (1.39–3.50)	
Complications, Clavien-Dindo score			0.699^c^
0	12.5 (n = 1)	7.1 (n = 1)	
I	12.5 (n = 1)	35.7 (n = 5)	
II	50.0 (n = 4)	50.0 (n = 7)	
IIIa	25.0 (n = 2)	7.1 (n = 1)	
No. pelvic lymph nodes identified on pathology			0.629^b^
Median (IQR)	6.5 (6.0–12.5)	6.0 (3.75–9.0)	
No. positive pelvic nodes			0.729^b^
Median (IQR)	1.5 (1.0–3.8)	2.0 (1.0–3.0)	
Distribution of positive pelvic nodes			0.667^c^
0	12.5 (n = 1)	7.1 (n = 1)	
1	37.5 (n = 3)	35.7 (n = 5)	
> 1	50.0 (n = 4)	57.1 (n = 8)	
Total positive regional lymph nodes (pelvic + inguinal)			0.821^b^
Median (IQR)	2.5 (1.25–3.75)	3.0 (1.0–7.0)	
Largest metastatic focus (mm)			0.623^b^
Median (IQR)	45.0 (25.0–72.0)	30.0 (19.8–65.0)	
Missing	0	3	
Extracapsular spread			0.965
Present	37.5 (n = 3)	38.5 (n = 5)	
Absent	62.5 (n = 5)	61.5 (n = 8)	
Missing	0	1	
Time to adjuvant therapy, weeks			0.018^b^
Median (IQR)	11.6 (10.6–18.5)	7.71 (6.29–10.4)	

Values are percentages unless otherwise indicated. ^*^Includes five patients in the oPLND group and ten patients in the rPLND group. ^†^Excludes two hospital stays of 41 days and 21 days in rPLND group due to nonsurgical factors detailed in Results. ^a^χ^2^ test; ^b^unpaired *t* test; ^c^χ^2^ for trend test

**Fig. 2:**
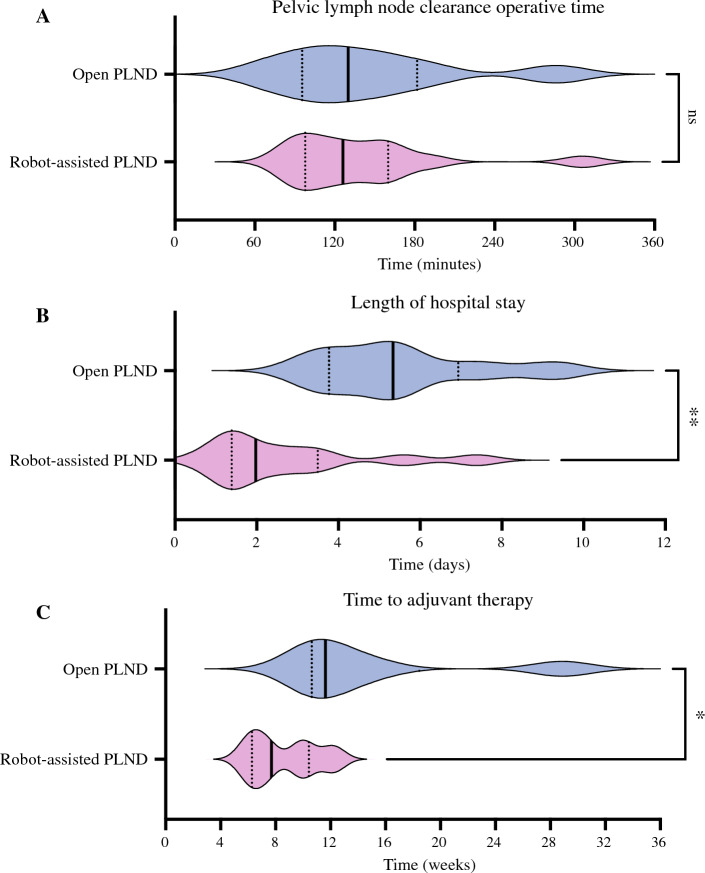
Improvements in post operative recovery and interval to starting adjuvant therapy with robot assisted PLND without change in operative duration compared to open PLND. **A** Distribution of operative time for pelvic node dissection (median open PLND 130 min (IQR 95*.*5–182) vs. for robot-assisted PLND 126 min (IQR 97*.*8–160), *p* = 0.836. **B** Distribution of length of hospital stay for open PLND (median open PLND 5.34 days (IQR 3.77–6.94) versus for robot-assisted PLND 1.98 days (IQR 1.39–3.50), *p* = 0.006). **C** Distribution of interval between surgery and start of systemic therapy (median open PLND 11.6 weeks (IQR 10.6–18.5) vs. for robot-assisted PLND 7.71 months (IQR 6.29–10.4), *p* = 0.018)

There was no statistically significant difference in median operating time for either pelvic dissection alone or for total operative time between treatment cohorts (pelvic dissection time median oPLND 130 min vs. rPLND 126 min, *p* = 0.836) (Table 2; Fig. 2). However, the patients undergoing a superficial groin lymph node dissection had a significantly shorter groin dissection in the oPLND group compared with the rPLND group (median groin dissection time oPLND 72 min vs. 111 min rPLND, *p* = 0.036). The reasons for this are unclear. The only difference between the two groups is that the groin dissection was performed before the pelvic dissection in the oPLND group but after pelvic dissection in the rPLND group.

Two patients in the rPLND group had prolonged surgical admission for nonsurgical issues (86-year-old with exacerbated postoperative delirium and needing nursing home placement due to living alone, and a 75-year-old with complex psychological needs with hospital stays of 41 days and 21 days, respectively). Excluding these two patients, hospital length of stay was significantly shorter in the rPLND group compared with the oPLND group similar to other published work (in-patient hospital length of stay median oPLND 5.34 days vs. rPLND 1.98 days, *p* = 0.006) (Table 2; Fig. 2).^9^ The length of perioperative stay in hospital was not significant for the entire cohort without these two exclusions (oPLND group 5.34 days vs. rPLND 2.48 days, *p* = 0.754). This improved recovery time from the surgery was additionally reflected in a significantly shorter interval to start adjuvant therapy (median oPLND 11.6 weeks vs. rPLND 7.71 weeks, *p* = 0.018) (Table 2). Adjuvant radiotherapy was given to two of the eight oPLND cases (25.0%) and two of the 14 rPLND cohort (14.3%).

### Melanoma-related Outcomes

**Fig. 3:**
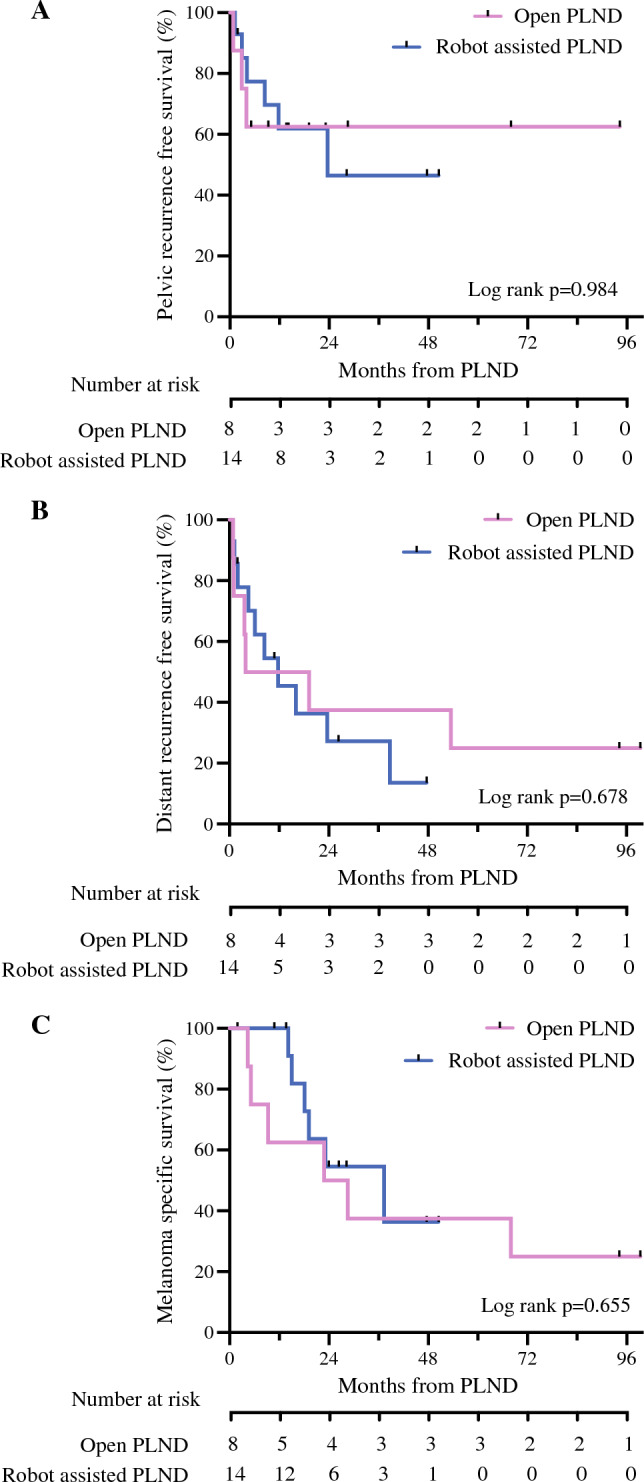
Kaplan-Meier survival estimates following pelvic lymph node dissection with either an open approach or robot-assisted surgery. **A** Pelvic lymph node basin recurrence-free survival (*p* = 0.984). **B** Distant recurrence-free survival (median survival open PLND 11.6 months vs. robot-assisted PLND 11.8 months, *p* = 0.678). **C** Melanoma-specific survival (median survival open PLND 25.7 months vs. robot-assisted PLND 37.4 months, *p* = 0.655, log-rank test)

There was a median follow-up of 25.7 (interquartile range [IQR] 6.16–87.7) months in the oPLND group, which was similar to the 21.1 months follow-up in the rPLND group (IQR 14.0-30.5 months) (Table 3). There were no differences in the pelvic in-basin lymph node recurrence-free survival, distant melanoma recurrence-free survival, or the melanoma-specific survival between the oPLND or rPLND groups (Fig. 3). Patterns of recurrence reflected in the numbers of no recurrence, in-basin lymph node recurrence, or distant site as first presentation of progression were similar between the two groups. Additionally, the numbers of lines of subsequent therapies required on progression was similar irrespective of surgical approach (Table 3).

**Table 3:** Patterns of melanoma recurrence grouped according to the method of pelvic lymph node dissection (open or robot-assisted)

	oPLND (*n* = 8) (2012–2016)	rPLND (*n* = 14) (2017–2023)	*P*^a^
Median follow-up, months			
Median	25.7 (6.16–87.7)	21.1 (14.0–30.5)	0.137^b^
Systemic therapy			
Therapy before PLND	37.5 (n = 3)	53.8 (n = 7)	0.571
Therapy after PLND	75.0 (n = 6)	92.9 (n = 13)	0.240
Lines of post op systemic therapy			0.588
None	25.0 (n = 2)	7.1 (n = 1)	
Adjuvant therapy	37.5 (n = 3)	57.1 (n = 8)	
Multiple (adjuvant and on progression)	37.5 (n = 3)	35.7 (n = 5)	
Melanoma recurrence			0.836^c^
Distant recurrence	75.0 (n = 6)	71.4 (n = 10)	
Pelvic recurrence	37.5 (n = 3)	42.9 (n = 6)	
No recurrence	12.5 (n = 1)	14.3 (n = 2)	

^a^χ^2^ test; ^b^unpaired *t* test; ^c^χ^2^ for trend test

## Discussion

The overall landscape of indications for pelvic lymph node dissection in melanoma has evolved. Completion lymph node dissection in the presence of microscopic sentinel lymph node involvement is no longer standard of care.^1,2^ Specifically in the Sunbelt melanoma trial, there were no survival benefits to performing a pelvic node dissection in addition to a completion inguinal lymph node dissection for microscopic melanoma metastasis to the inguinal sentinel lymph nodes.^19^ It was hoped that the EAGLE FM prospective, randomized, controlled trial (NCT02166788) would be able to further clarify the role of a completion pelvic lymph node dissection, but it has been difficult to recruit, with only 101 patients recruited to an accrual target of 634.^20^ The main surgical indication for pelvic lymph node dissection in melanoma is now a therapeutic lymph node dissection in the absence of other distant metastatic disease.

Robot-assisted pelvic lymph node dissection is an integral part of the oncological management of several cancers but is most commonly reported in the management of prostate cancers.^21^ Here, standard PLND has reported complication rates of 8.2%, with more extended lymph node templates including lymph node dissections over the iliac vessels increasing this to 19.8%.^22^ rPLND in melanoma for mixed indications have previously been reported to be well tolerated with low rate of surgical complications.^9^ The number of lymph nodes identified on surgical specimens from the pelvis depends on the extent of surgical dissection and histopathology identification but has been reported to be a median of 12 lymph nodes with an open pelvic dissection technique in melanoma.^23^ This number can be doubled with more extensive pelvic dissection techniques reaching the aortic bifurcation in a robot-assisted technique but comes at the cost of increasing complications.^24,25^ The median number of pelvic lymph nodes removed in our study is six, but more than 50% of dissections involved matted lymph nodes, making a direct comparison difficult and underestimates the number of lymph nodes truly removed in our cohort. There have been rare case reports of peritoneal carcinomatosis following robot-assisted prostatectomy and pelvic lymph node dissection, but no patients in our cohort had peritoneal recurrence.^26^

The long-term melanoma outcomes in this group of patients with macroscopically enlarged pelvic lymph nodes has not been separately discussed. Durable response has been reported following an aggressive surgical dissection and without effective systemic therapies.^27^ Similarly, the use of adjuvant systemic therapies in patients with melanoma in mixed cohorts of macroscopic and at-high-risk-for microscopic melanoma has shown comparable responses irrespective of oPLND or rPLND.^9^

Our study has some limitations given its retrospective cohort nature, small numbers, and study period spanning the advent of several systemic therapies. These limitations may underestimate complications, introduce type II errors, and represent differences in treatment schedule, but it is the largest series of pelvic dissections compared in modern melanoma management. Additionally, this is a comparison between a historical cohort (oPLND) and a more contemporary patient group (rPLND), which may introduce observation biases, although the follow-up period is similar across the groups. A prospective trial in patients with macroscopically enlarged melanoma lymph nodes is not likely to be possible given the application of effective adjuvant therapies after sentinel lymph node biopsy and is reflected in the slow accrual rate in the EAGLE FM study.^20,28,29^ Despite the possibility of inadvertent selection bias, our patient cohort remains relatively homogenous in its characteristics and detailed data available for all therapies applied over time.

The success of modern adjuvant targeted therapy and immunotherapy in resected Stage III melanoma has resulted in absolute clinical benefits for recurrence-free survival. Four key trials have led to several licensed drugs in Stage III melanoma (COMBI-AD Dabrafenib+Tramatenib relapse vs. placebo, hazard ratio [HR] 0.51; CheckMate238 Nivolumab relapse vs. Ipilumimab HR 0.72; Keynote054 Pembroluzimab relapse vs. placebo, HR 0.61; and SWOG1404 Pembroluzimab relapse vs. IFNα-2b or Ipilumimab, HR 0.77).^4–7^ This durable response has been transformational in the management of patients with macroscopically enlarged lymph nodes where the availability of an effective means of improving disease-specific survival without increasing morbidity. However, bulky pelvic lymph nodes are a concern in this context, especially when an oPLND is planned, as the time to recover from surgery could delay the start of systemic therapy leading to a potential failure of treatment. We show that reducing the start of adjuvant treatment in this group of high-risk melanoma patients by >3 weeks using rPLND does not influence nodal recurrence or melanoma-specific survival outcomes.

The timing of adjuvant immunotherapy after surgery remains incompletely understood. A recent publication has investigated the outcomes in Stage III melanoma following adjuvant therapy grouped in time intervals of <6 weeks, 6–12 weeks, and >12 weeks, which saw no compromise in outcomes when adjuvant immunotherapy was initiated beyond 6 weeks from resection.^30^ This is difficult to interpret directly in the context of macroscopic pelvic lymph nodes as the publication includes a broad cohort of patients undergoing adjuvant therapy in melanoma for both microscopic disease (SLNB identified) and macroscopic disease (TLND identified) for low tumor burden. In the two cohorts presented, 172 of 626 (27.5%) and 1392 of 3712 (38.6%) were following a therapeutic lymph node biopsy at all lymph node basins.^30^ We believe that pelvic lymph nodes are especially challenging as complete surgical clearance is more technically challenging compared with the other large lymph node basins, and no subgroup analysis is done to directly compare outcomes in this high-risk group based on interval to adjuvant therapy after surgery. More data are required to understand whether timing of adjuvant therapy influences outcomes in the group of highest risk in patients with enlarged pelvic lymphadenopathy.

More recently, there has been success in managing patients with Stage IV and irresectable Stage III melanoma with a neoadjuvant approach using immunotherapy demonstrated in the OpACIN, OpACIN-neo, and PRADO trials.^31–33^ These trials have collectively demonstrated that neoadjuvant immunotherapy in patients with macroscopic nodal disease have better recurrence-free survival with fewer adverse effects, to the extent that where a complete pathological response is seen in the lymph node dissection specimen additional postsurgical immunotherapy does not add benefit at 2-year endpoint analysis. This has led to the ongoing NADINA trial comparing response driven neoadjuvant combination of ipilimumab and nivolumab versus adjuvant nivolumab (NCT04949113).^34^

Given the poor outcomes associated with macroscopically enlarged pelvic nodes, the demonstrable benefits of a neoadjuvant approach to macroscopic lymph node management in melanoma and the good recovery associated with rPLND, surely the time has come for neoadjuvant therapy in this group of patients?
